# Sex Differences in Circulating T-Tau Trajectories After Sports-Concussion and Correlation With Outcome

**DOI:** 10.3389/fneur.2020.00651

**Published:** 2020-07-07

**Authors:** Stefania Mondello, Vivian A. Guedes, Chen Lai, Andreas Jeromin, Jeffrey J. Bazarian, Jessica M. Gill

**Affiliations:** ^1^Department of Biomedical and Dental Sciences and Morphofunctional Imaging, University of Messina, Messina, Italy; ^2^National Institutes of Health, National Institute of Nursing Research, Bethesda, MD, United States; ^3^Cohen Veterans Biosciences, Cambridge, MA, United States; ^4^University of Rochester School of Medicine and Dentistry, Rochester, NY, United States

**Keywords:** sports-related concussion (SRC), t-tau, sex differences, biomarker, brain injury- traumatic, outcome, return to play

## Abstract

Sex differences in molecular biomarkers after sports-related concussion (SRC) could steadily advance our understanding of injury heterogeneity and complexity, and help capture phenotypic characteristics, by unveiling sex-dependent pathobiological processes and disease mechanisms. Such knowledge will help improve diagnosis, clinical management, and prognosis. Total-tau (t-tau) has recently emerged as a promising blood marker showing sex-associated differences in neurodegenerative diseases. Nonetheless, to date, little is known about the potential influence of sex on its injury-related concentration and dynamics after SRC. We hypothesized that measurements of circulating levels of t-tau over time would reflect a differential vulnerability signature, providing insights into the sex-related phenotypes and their relationship with clinical outcomes. To test this hypothesis, plasma levels of t-tau were measured using an ultrasensitive immunoassay up to 7 days after injury, in 46 concussed athletes (20 males, 26 females). We used trajectory analysis to generate two distinct temporal profiles of t-tau, which were then compared with gender and return to play (RTP). The majority of subjects (~63%) started with low t-tau concentrations that further declined within the first 48 h; while the remaining (“maximal decliners”) started with concentrations comparable to the baseline levels that also fell over time, but persisting markedly higher compared with the first profile. The maximal decliner group was primarily composed of female subjects (*p* = 0.007) and was significantly associated with poor outcome (RTP ≥ 10 days after concussion) (*p* = 0.011). Taken together, our data provide evidence for the existence of sex-related biosignatures following sports-related concussions, possibly indicating a differential effect as a result of distinct brain vulnerability and inherent injury response. Future studies will be required to further elucidate underlying sex-based biological and pathophysiological mechanisms, and determine the value of t-tau signatures for management and therapeutic decision-making in sports-related concussions.

## Introduction

Research on sport-related concussion (SRC) has increased dramatically in recent years, owing to its daunting burden—estimates suggest that up to 3.8 million cases occur annually, in the US alone—and potential for severe long-term consequences (1, 2). Contextually, sex-based differences have been examined suggesting distinct sequalae and outcomes in male and female athletes, possibly requiring a tailored approach to clinical management (3). Hence, sensitive and objective tools capable of providing new insights into the unique aspects of sex-dependent biological mechanisms and identifying associated phenotypes are vital to inform future research and therapeutic advances.

Several candidate biomarkers have emerged as potential blood tests to aid concussion management. Aside from objectively assessing SRC presence, such markers have been suggested to predict and monitor recovery, while advancing our understanding of the underlying pathophysiological mechanisms of concussion (4–7). Nonetheless, there has been a preponderant focus on male athletes, and very few studies have explored sex-related variability of brain injury biomarkers and their relationships with the underlying neuropathological and clinical characteristics of SRC (8, 9). This lack of biological data and the ensuing knowledge gap of the molecular drivers and processes associated with sex disparities following sports-concussion is a major obstacle to biomarker clinical translation.

Clinical studies corroborated by epidemiological evidence in neurodegenerative diseases have suggested a role that sex may play in modulating the release of tau, a neuroaxonal injury marker, into biofluids while interacting with disease development and progression (10–12). Moreover, the use of an ultra-sensitive assay—single molecule array (SIMOA) technology—has consistently demonstrated the feasibility and reliability of detecting substantially low concentrations of total-tau (t-tau) in blood, making possible its accurate longitudinal measurement in athletes (5, 13–15). Thus, the current work investigated the potential influence of sex in the expression and release of t-tau (16), following sports-related concussion. More specifically, using SIMOA technology, we evaluated temporal profiles for t-tau in a population of concussed and non-concussed athletes in relation to sex and outcome. We hypothesized that the identification of sex-related biomarker signatures could improve phenotype characterization and help elucidate the biological and pathophysiological basis of SRC.

## Methods

### Participants Selection and Assessment

This research is part of a prospective study on concussion conducted among collegiate Athletes from the National Collegiate Athletic Association (NCAA) division I and III (5). Here, we report on a group of athletes (male 41 and female 42) from soccer (*n* = 38), American football (*n* = 26), basketball (*n* = 8), hockey (*n* = 4), and lacrosse (*n* = 4), selected from a cohort of 632 NCAA participants accrued between 2009 and 2014 (Table 1).

**Table 1:** Characteristics of the 83 athletes included in the study.

		**Athletes** **(*n =* 83)**	**Male** **Athletes** **(*n =* 41)**	**Female** **athletes** **(*n =* 42)**	***P*-value**
Age, yrs		18.9 ± 0.97	18.92 ± 1.14	18.90 ± 0.79	0.96
Gender	Male	41 (49.4%)			
Race	White	58 (69.9%)	29 (70.73%)	29 (69.05%)	0.33
	African American	3 (3.6%)	3 (7.32%)	–	
	Asian	–	–	–	
	More than one race	4 (4.8%)	2 (4.88%)	2 (4.76%)	
	Unknown	18 (21.7%)	7 (17.07%)	11 (26.19%)	
Ethnicity	Non-Hispanic or Latino	36 (43.4%)	13 (31.71%)	23 (54.76%)	0.05
	Latino or Hispanic	1 (1.2%)	1 (2.44%)	-	
	Unknown	46 (55.4%)	27 (65.85%)	19 (45.24%)	
Sport	Soccer	38 (45.8%)	8 (19.51%)	30 (71.42%)	**<0.0001**
	Football	29 (35%)	29 (70.73%)	-	
	Basketball	8 (9.6%)	2 (4.88%)	6 (14.29%)	
	Hockey	4 (4.8%)	2 (4.88%)	2 (4.76%)	
	Lacrosse	4 (4.8%)	–	4 (9.53%)	
Concussion	Yes	46 (55.4%)	26 (63.41%)	20 (47.62%)	0.15
	No	37 (44.6%)	15 (36.59%)	22 (52.38%)	
Prior Concussions	0	31 (72%)	17 (73.91%)	14 (80%)	0.95
	1	7 (16.3%)	3 (13.04%)	4 (20%)	
	2	3 (7%)	2 (8.70%)	1 (5%)	
	3	2 (4.7%)	1 (4.35%)	1 (5%)	
	Missing	3			
RTP, days, median (IQR)		11 (6–17) (Range 2–138)	7 (5–15.5)	13 (11–21)	**0.01**

*Data are presented with n (%), mean (±SD), or median (IQR) in case of non-normal distribution. RTP, Return to play; NA, Not Applicable. The bold values indicate p < 0.05*.

Enrolled subjects underwent blood sampling and cognitive testing prior to the sports season, and were followed prospectively for a diagnosis of SRC. To avoid a missed diagnosis of concussion, an on-field certified athletic trainer was present at every game and defined SRC according to diagnostic guidelines on sports-related concussion (17). The severity of SRC was graded based on the resolution of concussion symptoms (i.e., number of days it takes for a player to return to play [RTP]) into short (<10 days) and long (≥10 days) RTP (14). The return-to-play decision was determined by athletic trainers or team physicians at their respective universities and was based on the NCAA best practices (http://www.ncaa.org/sport-science-institute/concussion-diagnosis-and-management-best-practices). In players who sustained SRC, consecutive blood samples were collected within 6 h of injury (median 1.6 h), and at 2, 3, and 7 days post-injury. For the follow-up time points, blood samples were collected between 9 and 10 AM under non-fasting conditions. Plasma sampling at the same time points as SRC athletes was also performed in non-concussed teammate athletes, who did not significantly differ in sport played, history of SRC, or any other demographic feature and served as controls.

The study was approved by the Institutional Review Board at the University of Rochester and Rochester Institute of Technology (approval protocol numbers: 24457 and 22971). Written informed consent was obtained from all participants before enrollment.

### Blood Collection and Biomarker Analysis

Venous blood was collected in a non-fasting state by venipuncture into EDTA tubes and placed on ice until processed. All blood was centrifuged within 60 min from the time of blood draw, at 4°C at 3,000 rpm for 10 min. Then plasma was separated, aliquoted, and stored at−80°C pending analysis.

T-Tau concentrations in plasma samples were measured by an ultrasentive immunoassay using SIMOA technology (Quanterix Corporation, Lexington, MA), a digital form of ELISA (13, 18). The Simoa human t- tau assay is based on a sandwich antibody complex that reacts with an epitope in the midregion of the molecule and recognizes all tau isoforms. Two quality control (QC) samples at low and high concentration of the respective analyte were used for assay quality assurance and to assess the overall precision. The limit of detection for the assay is 0.012 pg/mL. The average intra-assay duplicate coefficient of variation was 8.25% (SD <10%). Results were reported in picograms/milliliter (pg/mL). Samples were analyzed at the same time in duplicates and using the same batch of reagents by trained laboratory technicians who were blind to demographic and clinical information.

### Statistical Analysis

Statistical analyses were conducted using Stata Data Analysis and Statistical Software (v.13, College Station, Texas). Baseline characteristics were summarized using standard descriptive statistics, and an exploratory analysis was carried out to determine the distribution of the demographic and clinical variables. Continuous variables are presented as mean (SD) or median (interquartile range [IQR]), and categorical variables are summarized as absolute frequencies and percentages. To identify differences between groups in biomarker concentrations, Mann-Whitney U and Wilcoxon signed-rank tests were applied, as appropriate. The non-parametric Friedman test, followed by *post-hoc* pairwise multiple comparisons (Dunn's test) was performed to evaluate biomarker changes over time. Group-based trajectory analysis (TRAJ) was used to explore biomarker levels in blood using the Stata program and to identify clusters of individuals following trends over time. The TRAJ procedure determines patterns in longitudinal biomarker data by assuming that the population is composed of distinct subgroups containing their own unique biomarker profiles and can handle data that is missing completely at random (19, 20). The trajectories are identified on a likelihood basis using methods previously described (21–23). A censored normal model was used given the minimal detectable limit for each biomarker and the skewed distribution. The number of distinct trajectories for each biomarker was determined by using a combination of the Bayesian information criterion (BIC), Akaike information criterion (AIC), and clinical judgment. Specifically, while the clinical knowledge guided the decision on the maximum number of plausible groups, BIC and AIC were used as the criteria for model selection, which was also moderated by the rule of parsimony (i.e., selecting the simplest model that best describes the data). The final model captured the essential features of the data in the most comprehensible, parsimonious, and analytically tractable manner. Bivariate analyses were performed to explore the TRAJ group associations with sex and outcome. A contingency table was constructed to determine sensitivity and specificity. All tests were two-sided, and significance was determined at *p* < 0.05.

## Results

### Description of Population

Eighty-three (41 male and 42 female) athletes were included in the study. The average age was 18.9 years (SD, 0.97 years; range 18–23 years), and over two-thirds (69.9%) of participants were white. Baseline demographic characteristics did not differ between female and male athletes (Table 1). Female athletes were more likely than their male counterpart to play soccer (30 [71.42%] vs. 8 [19.51%]) and basketball (6 [14.19%] vs. 2 [4.88%]), while the vast majority of male participants were football players (29 [70.73%]). Forty-six subjects (55.4%) suffered a concussion, and twelve (28%) of them had a prior history of concussion. No significant differences were seen between males and females in regard to the occurrence of concussion and previous exposure, though, symptoms in female athletes lasted longer (median 7 vs. 13 days, *p* = 0.01).

### Blood Levels and Longitudinal Changes of T-Tau in Male and Female Athletes

**Figure 1:**
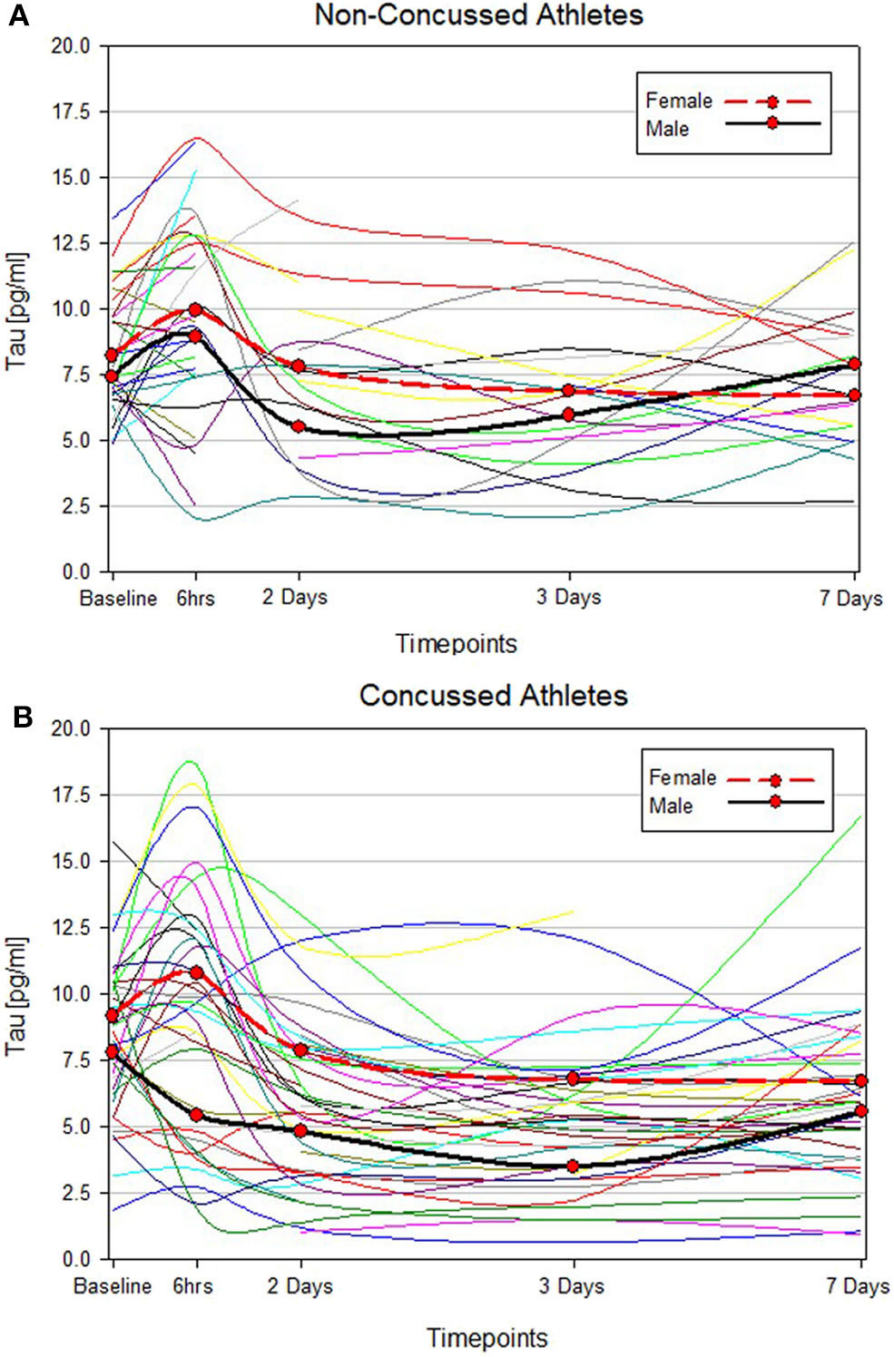
Individual t-tau time course profiles in control and concussed athletes. The spline curves represent the time course of t-tau in non-concussed **(A)** and concussed **(B)** study participants. The 2 bold lines represent median values of t-tau in male (black) and female (red).

T-tau results in men and women were compared both at baseline and longitudinally. We found no significant difference between men and women with respect to the blood levels of t-tau at baseline (*p* = 0.4). Among non-concussed athletes, there were no substantial changes in t-tau over time in either men or women, and we found no differences between sexes (Figure 1A, Supplementary Table 1). Conversely, among concussed athletes, we found an altered temporal profile of plasma t-tau following injury in both sexes. In men, plasma t-tau concentration substantially decreased at day 2 (*p* < 0.01 vs. baseline), with the lowest concentrations being measured in samples collected 3 days post-SRP (2.2-fold decreased compared to baseline, *p* < 0.001). In women, after an initial, but not significant, increase at the 6 h time point, plasma t-tau concentrations dropped on day 2 and remained stably low throughout the study period. Nonetheless, plasma t-tau after SRC was consistently higher in female than male athletes up to 3 days after injury, while the highest differences were observed at 6 h and 3 days after SRC (10.78 vs. 5.42 pg/ml, *p* = 0.017, and 6.78 vs. 3.49 pg/ml, *p* = 0.0006, respectively), and returned to comparable levels only on day 7 (Figure 1B, Supplementary Table 1).

### Trajectory Profiles of Plasma T-Tau in Concussed Players

**Figure 2:**
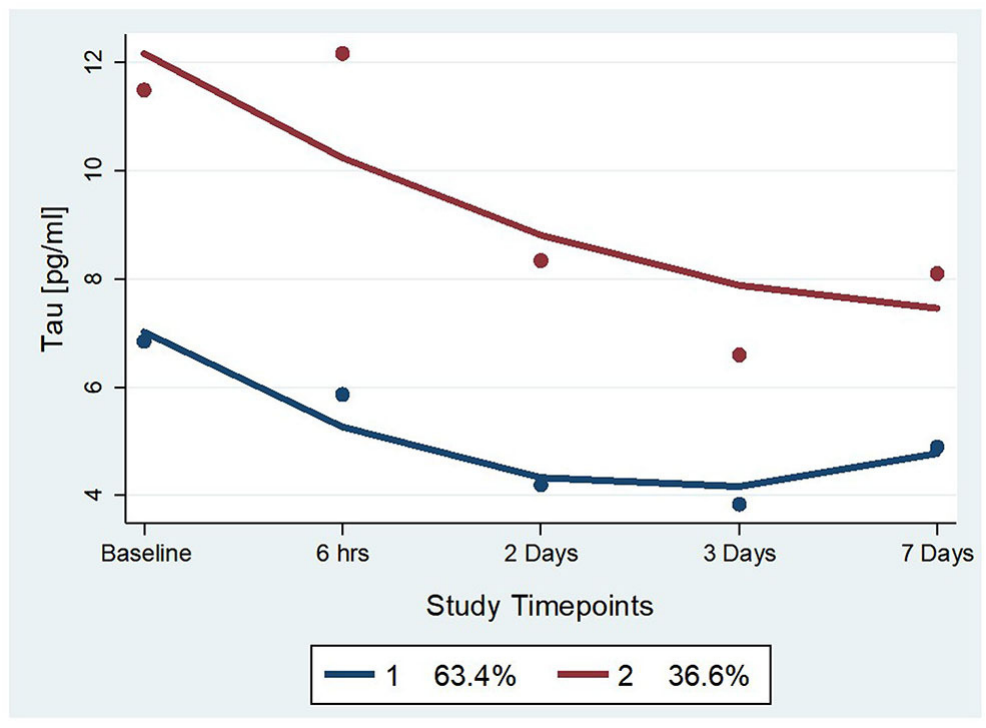
Trajectory groups for profiles over time and percent membership for each trajectory group for serum t-tau. The group-based trajectory analysis (TRAJ) procedure identified 2 groups. The “Low Transient Decliners” group (blue line) included 63% of the subjects. These were subjects with initially low concentrations of plasma t-tau, which further decreased over time. The “Maximal Decliners” group (red line) included the remaining 37% of the subjects, who showed a similar temporal pattern but with consistently higher levels of t-tau.

Two statistically distinct temporal profiles were identified for tau as the best model by trajectory analysis (TRAJ) (Figure 2). T-tau concentrations in both groups decreased over time after concussion. However, while one group (“low transient decliners”), which includes the majority of subjects (~63%), started with low concentrations of t-tau that more substantially declined between day 2 and 3 to slightly rose again on day 7; the other group (“maximal decliners”) started with concentrations comparable to the baseline levels that decreased over time, albeit, remained markedly higher compared with the other group (Figure 2).

Tau TRAJ group comparisons were made with concussed athlete features. There were differences by tau TRAJ group membership with regard to gender and RTP, but not concerning the other demographic and clinical variables. The maximal decliner group was associated with female gender and RTP equal to or more than 10 days after concussion (Table 2). The sensitivity and specificity for the prediction of high RTP with the 2-group model were 57 and 78%, respectively (Table 3).

**Table 2:** Bivariate trajectory group associations with demographic and clinical variables after concussions.

		**Low Transient Decliners** **(*n =* 28)**	**Maximal decliners** **(*n =* 18)**	***P*-value**
Gender	Female, *n* (%)	8 (28.6%)	12 (66.7%)	*P* = 0.007
RTP	RTP (≥10 days), *n* (%)	10 (35.7%)	13 (72.2%)	*P* = 0.011

**Table 3:** Contingency table of 2-group Tau model for predicting high RTP.

	**Low RTP (*n =* 23)**	**High RTP (*n =* 23)**
Maximal Decliners	5 False Positive	13 True positive
Low Transient Decliners	18 True Negative	10 False Negative
	Specificity 78% (18/23)	Sensitivity 57% (13/23)

## Discussion

In this longitudinal study, we explored the effect of biologic sex on circulating t-tau in healthy collegiate athletes and following sports-related concussions. While there were no between-sex differences in blood t-tau levels at baseline and after normal activity, indicating that no adjusted thresholds are needed to be adopted for clinical use in young adult athletes, we show altered concentrations and dynamics in both sexes after concussion; with a distinct temporal profile and substantially higher levels of t-tau in concussed female athletes compared to their male counterparts. Taken together, these findings suggest a sex-specific biologic response of the brain to SRC.

Why circulating t-tau substantially decreases following sports-concussion is unexplained. *In vitro* and animal studies have shown that neuronal and synaptic activity dynamically regulate the active secretion of tau into the extracellular space (24). Thus, a potential interpretation could be that normal secretion of tau from neurons into brain interstitial fluid is compromised following sports-concussion, especially in men. Mechanical injury may possibly trigger intraneuronal accumulation and missorting of tau, leading to abnormal phosphorylation and tau truncation (25), which, in turn, results in tau-dependent neuronal malfunction and atrophy and reduced circulating levels (26). On the other hand, several mechanisms could underlie the observed sex difference in t-tau. These include the hormonal asset that may directly affect tau hyperphosphorylation (27). A second possibility is that sex interacts with genetic risk factors (e.g., the apolipoprotein E [APOE] genotype) to drive a distinct downstream response to SRC, including different mechanisms of intra- and trans-cellular mechanisms of tau sorting and exosome biogenesis and spreading (11, 12, 28). Moreover, additional reasons for the disparity observed in t-tau levels could lie in the marked sexual dimorphism in the brain organizational and connectivity patterns, cerebrovascular function, and the post-injury inflammatory responses, which may play a primary or contributing role in characterizing and determining sex-specific SRC pathophysiology and phenotypes (29–31).

Interestingly, no studies to date have identified female athletes with chronic traumatic encephalopathy (CTE) (32). It is, therefore, tempting to speculate that the more marked release of t-tau into the blood of female athletes following sports-concussion could in some extent exert a protective role by preventing brain interstitial accumulation (i.e., neurofibrillary tangles) (33), thereby entailing that the risk for neurodegeneration is modulated in a sex-specific manner. While suggestive, these speculations need to be investigated in future studies specifically targeting the clearance pathways, including the exosome profile, of endogenously produced injury markers after SRC. Nevertheless, our work suggests that sex differences in t-tau may present a powerful key to understanding the biological basis and mechanistic links between traumatic brain injury and pathogenesis of neurodegenerative processes and diseases.

Based on our characterization of the plasma kinetics of t-tau following sports-concussion, substantial differences in t-tau between male and female athletes were measured between 6 and 3 days following injury, but not after (7 days), pointing out a specific time window for investigating sex-based mechanisms underlying SRC. From a pathophysiological perspective, the female early monophasic rise compared to the male delayed monophasic decrease may reflect distinct immediate responses to initial insult as well as secondary injury mechanisms—axonal injury, induced tau hyperphosphorylation—or a combination of both. Future studies are required to explore whether the observed divergence in t-tau levels match distinct anatomical patterns, and if that is the case, their correlation with the extent of the injuries (34, 35).

The trajectory results show that the *maximal decliner* group is associated with both being female and worse outcomes, which may seem counterintuitive. These findings may perhaps be a function of the presence of APOE ε4 allele, given its interaction with tau pathogenesis and association with outcome after SRC (11, 36, 37). Future work should evaluate the genetic drivers of biomarker profile and cognitive impairment after SRC in a sex-specific manner to identify novel pathways of risk and promote safer sports play. It is also possible that the observed association reflects women's inherent neuroanatomical differences (38), leading to higher susceptibility to traumatic axonal injury and neuronal vulnerability. Such argument fits well with the hypothesis that axonal injury is the main determinant of long-term impairments following SRC, and is in line with a recent study reporting more widespread evidence—a 5-fold difference—of microstructural white matter alterations in female athletes following subconcussive repetitive trauma (35).

In regards to the clinical implications of the trajectory patterns, the *maximal decliner* group shows fairly high specificity (78%) for the identification of prolonged RTP and may be suitable for the recruitment of subjects into interventional trials. In contrast, the relatively modest sensitivity (57%), which may be partly explained by the fact that t-tau alone is unable to capture the complex pathobiology and ensuing sequelae of SRC, underpins the need for a multimarker strategy.

Our findings, while corroborating and complementing previous studies (39–43), link SRC with a sex biologic divergence of a circulating neuroaxonal injury marker, namely t-tau, demonstrating their association with adverse clinical outcomes-longer RTP. We interpret these results as emphasizing the need for increased awareness of sex-related SRC variability and clinical relevance, and for more extensive research to unearth and elucidate the neurobiological underpinnings of sex-differences in biomedicine, particularly following sports-concussions (44–46).

Limitations of this study include the overall modest sample size. Caution, therefore, is needed in interpreting our results until they can be confirmed in subsequent larger cohorts. Another limitation is that our examination was restricted to young adult athletes (age range 18–22 yrs). Future studies are required to carefully explore the effect of biological sex on circulating t-tau after SRC across the age spectrum. Such information could be particularly valuable in pediatric SRC to interpret the impact of insults during the various phases of brain development (47). Finally, we did not have advanced imaging data. Future work integrating neuroimaging parameters and a panel of novel pathobiologically diverse blood biomarkers (28, 48, 49) is a critical avenue of investigation as it is likely to enhance our understanding of the complex relationships between sex and brain injury following SRC, improve our ability to characterize sex-related phenotype, and deliver targeted and tailored interventions.

The impact of sex, a primary aspect of biological and pathological variability, has been underestimated in sports research and, particularly, related biomarker studies. To move the field forward, it is vital to identify drivers of sexual disparities through the identification of blood biomarkers of specific underlying pathobiological mechanisms. Ultimately, this knowledge will have a significant role and transformative potential in informing clinical trial design and guidelines, and for developing precision-based management and therapies of concussed athletes.

## Data Availability Statement

The datasets generated for this study are available on request to the corresponding author.

## Ethics Statement

The studies involving human participants were reviewed and approved by The institutional review board at the University of Rochester and Rochester Institute of Technology. The patients/participants provided their written informed consent to participate in this study.

## Author Contributions

SM was responsible for performing the statistical analysis and the interpretation of the data and wrote the first draft of the paper. VG and CL carried out the laboratory work, participated in data analysis and data interpretation, and contributed to final review and amendment of the manuscript. AJ participated in data analysis, contributed to data interpretation, and to the final review and amendment of the manuscript. JB was responsible for designing the project, participated in data collection and patient enrollment, contributed to the interpretation of data, and revised the manuscript for content. JG contributed to conceptualizing the study, supervised laboratory work, contributed to data interpretation, and revised the manuscript for intellectual content. All authors read and approved the article for publication.

## Conflict of Interest

The authors declare that the research was conducted in the absence of any commercial or financial relationships that could be construed as a potential conflict of interest.
