# Supplementary material for: Sex Differences in Circulating T-Tau Trajectories After Sports-Concussion and Correlation With Outcome
Source: Front Neurol. 2020 Jul 7;11:651. doi: 10.3389/fneur.2020.00651 (PMC7358531; doi:10.3389/fneur.2020.00651)
Supplement: Supplementary file 1 [file Table_1.DOCX]

**Supplementary Table S1. Plasma t-tau levels in the 83 athletes included in the study stratified by concussion status and sex.**

|  |  | **Non-Concussed Athletes**  **(n=37)** | | | |  | **Concussed Athletes**  **(n=46)** | | | |  |
| --- | --- | --- | --- | --- | --- | --- | --- | --- | --- | --- | --- |
|  | **Time points** | **N** | **Male** | **N** | **Female** | **p**  **value^a^** | **N** | **Male** | **N** | **Female** | **p**  **value^a^** |
| **t-tau [pg/ml]** | **Baseline** | 15 | 7.41  (6.87-10.8) | 22 | 8.21  (6.71-9.69) | NS | 26 | 7.78  (5.34-9.70) | 20 | 9.18  (6.56-10.96) | NS |
|  | **6 hrs** | 13 | 8.94  (6.24-13.6) | 18 | 9.95  (7.56-12.54) | NS | 24 | 5.42  (3.80-10.34) | 19 | 10.78  (8.04-14.47) | **0.017** |
|  | **2 Days** | 5 | 5.51  (3.84-10.39) | 12 | 7.81  (6.65-9.66) | NS | 24 | 4.81 ******  (2.93-6.13) | 20 | 7.86 ††  (5.54-8.66) | **0.01** |
|  | **3 Days** | 6 | 5.96  (4.02-8.39) | 11 | 6.87  (5.48-8.49) | NS | 21 | 3.49 ******* ††  (2.48-5.65) | 19 | 6.78 ****** †††  (4.89-7.27) | **0.0006** |
|  | **7 Days** | 6 | 7.88  (5.41-12.33) | 11 | 6.69  (5.6-8.99) | NS | 24 | 5.55  (3.94-6.5) | 18 | 6.70 ***** †††  (4.60-8.75) | NS |
| Data are given as median (IQR). NS= not significant  ^a^ *p* values of the Mann-Whitney test for differences between male and female athletes.  * (p < 0.05), **(p < 0.01) or ***(p < 0.001) compared to baseline. †† (p < 0.01) or ††† (p < 0.001) compared to 6 hours (Friedman test followed by Dunn's Multiple Comparison Test) | | | | | | | | | | | |
